# 2-(Naphthalen-1-yl)-*N*-(1,3-thia­zol-2-yl)acetamide

**DOI:** 10.1107/S1600536812031583

**Published:** 2012-07-18

**Authors:** Hoong-Kun Fun, Ching Kheng Quah, Prakash S. Nayak, B. Narayana, B. K. Sarojini

**Affiliations:** a X-ray Crystallography Unit, School of Physics, Universiti Sains Malaysia, 11800 USM, Penang, Malaysia; b Department of Studies in Chemistry, Mangalore University, Mangalagangotri 574 199, India; c Department of Chemistry, P. A. College of Engineering, Nadupadavu, Mangalore 574 153, India

## Abstract

In the title compound, C_15_H_12_N_2_OS, the naphthalene ring system [maximum deviation = 0.026 (1) Å] forms a dihedral angle of 85.69 (6)° with the thia­zole ring [maximum deviation = 0.010 (1) Å]. In the crystal, inversion dimers linked by pairs of N—H⋯N hydrogen bonds generate *R*_2_^2^(8) loops. The dimers are linked by C—H⋯O hydrogen bonds into chains propagating along [110].

## Related literature

For general background to and the related structures of the title compound, see: Fun *et al.* (2010 ▶, 2011*a* ▶,*b* ▶, 2012 ▶). For the stability of the temperature controller used for the data collection, see: Cosier & Glazer (1986 ▶).
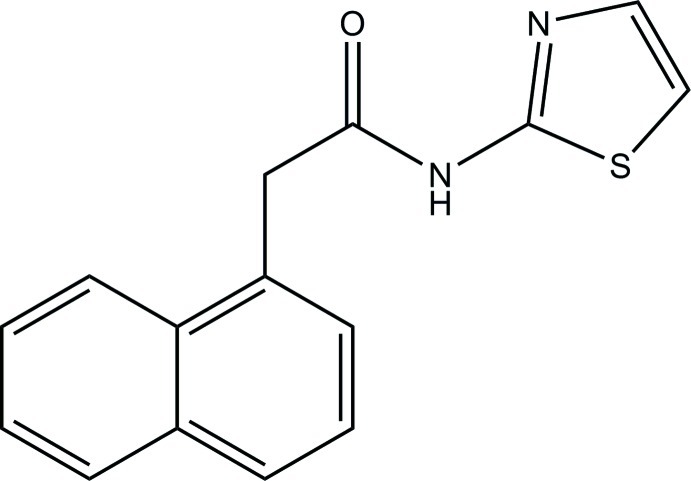


## Experimental

### 

#### Crystal data

C_15_H_12_N_2_OS
*M* *_r_* = 268.33
Monoclinic, 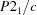

*a* = 5.2668 (1) Å
*b* = 13.0861 (2) Å
*c* = 18.5373 (3) Å
β = 105.640 (1)°
*V* = 1230.32 (4) Å^3^
*Z* = 4
Mo *K*α radiation
μ = 0.26 mm^−1^
*T* = 100 K
0.39 × 0.22 × 0.18 mm

#### Data collection

Bruker SMART APEXII CCD diffractometer
Absorption correction: multi-scan (*SADABS*; Bruker, 2009 ▶) *T*_min_ = 0.907, *T*_max_ = 0.956
17425 measured reflections
4470 independent reflections
3657 reflections with *I* > 2σ(*I*)
*R*_int_ = 0.036

#### Refinement

*R*[*F* ^2^ > 2σ(*F* ^2^)] = 0.047
*wR*(*F* ^2^) = 0.108
*S* = 1.06
4470 reflections
172 parameters
H-atom parameters constrained
Δρ_max_ = 0.48 e Å^−3^
Δρ_min_ = −0.28 e Å^−3^

### 

Data collection: *APEX2* (Bruker, 2009 ▶); cell refinement: *SAINT* (Bruker, 2009 ▶); data reduction: *SAINT*; program(s) used to solve structure: *SHELXTL* (Sheldrick, 2008 ▶); program(s) used to refine structure: *SHELXTL*; molecular graphics: *SHELXTL*; software used to prepare material for publication: *SHELXTL* and *PLATON* (Spek, 2009 ▶).

## Supplementary Material

Crystal structure: contains datablock(s) global, I. DOI: 10.1107/S1600536812031583/hb6894sup1.cif

Structure factors: contains datablock(s) I. DOI: 10.1107/S1600536812031583/hb6894Isup2.hkl

Supplementary material file. DOI: 10.1107/S1600536812031583/hb6894Isup3.cml

Additional supplementary materials:  crystallographic information; 3D view; checkCIF report

## Figures and Tables

**Table 1:** Hydrogen-bond geometry (Å, °)

*D*—H⋯*A*	*D*—H	H⋯*A*	*D*⋯*A*	*D*—H⋯*A*
N2—H1⋯N1^i^	0.85	2.07	2.9099 (16)	172
C13—H13*A*⋯O1^ii^	0.95	2.52	3.1929 (19)	128

Symmetry codes: (i) 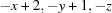; (ii) 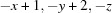.

**Fig. 1.**
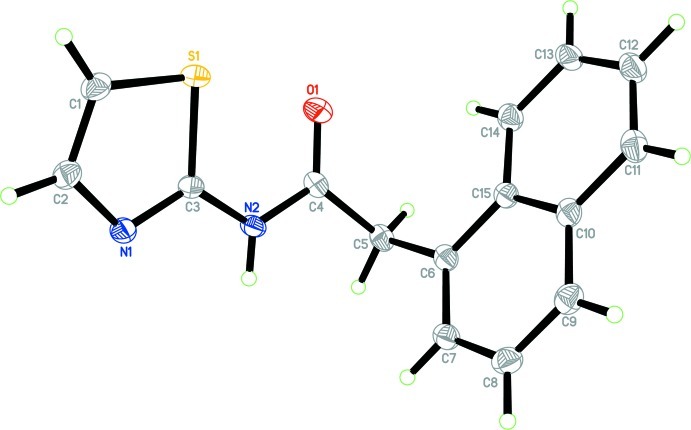
The molecular structure of the title compound showing 50% probability displacement ellipsoids for non-H atoms.

**Fig. 2.**
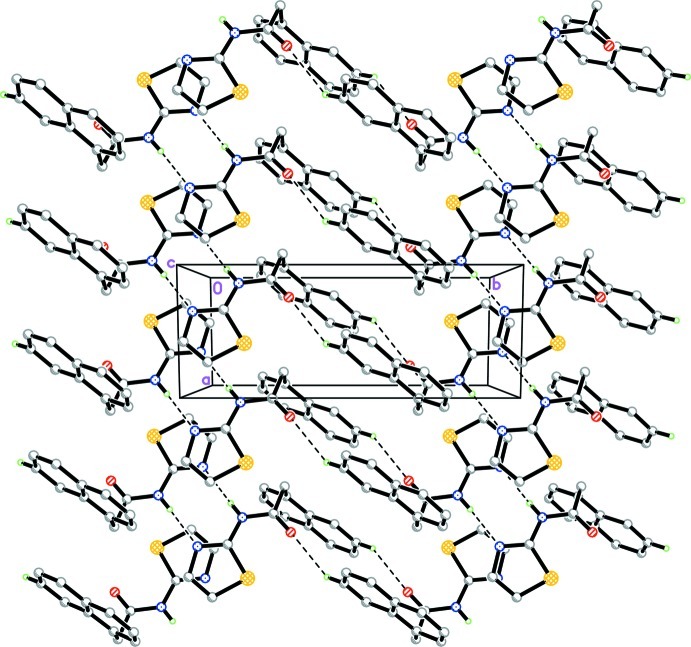
The crystal structure of the title compound, viewed along the c axis. H atoms not involved in hydrogen bonds (dashed lines) have been omitted for clarity.

## supplementary crystallographic information

### Comment

In continuation of our work on synthesis of amides (Fun *et al.*, 2010, 2011*a*, 2011*b*, 2012), we report herein the crystal structure of the title compound.

The molecular structure is shown in Fig. 1. Bond lengths are comparable to related structures (Fun *et al.*, 2010, 2011*a*, 2011*b*, 2012). The naphthalene ring system (C6-C15, maximum deviation of 0.026 (1) Å at atom C6) forms a dihedral angle of 85.69 (6)° with the thiazol-2-yl ring (S1/N1/C1-C3, maximum deviation of 0.010 (1) Å at atom C3).

In the crystal structure, Fig. 2, molecules are linked *via* N2–H1···N1 and C13–H13A···O1 hydrogen bonds (Table 1) into one-dimensional chains along [110].

### Experimental

1-Naphthalene acetic acid (0.186 g, 1 mmol), 2-aminothiazole (0.1 g, 1 mmol) and 1-ethyl-3-(3-dimethylaminopropyl)-carbodiimide hydrochloride (1.0 g, 0.01 mol) were dissolved in dichloromethane (20 mL). The mixture was stirred in presence of triethylamine at 273 K for about 3 h. The contents were poured into 100 mL of ice-cold aqueous hydrochloric acid with stirring, which was extracted thrice with dichloromethane. Organic layer was washed with saturated NaHCO_3_ solution and brine solution, dried and concentrated under reduced pressure to give the title compound (I). Brown blocks were grown from methanol and dichloromethane (1:1) mixture by the slow evaporation method (*m. p.* : 475-477 K).

### Refinement

The N-bound hydrogen atoms was located in a difference Fourier map and refined using a riding model *U*_iso_(H) = 1.2 *U*_eq_(N) [N–H = 0.8458 Å]. The remaining H atoms were positioned geometrically and refined using a riding model with C–H = 0.95 or 0.99 Å and *U*_iso_(H) = 1.2 *U*_eq_(C).

### Crystal data

**Table:**

C_15_H_12_N_2_OS	*F*(000) = 560
*M**_r_* = 268.33	*D*_x_ = 1.449 Mg m^−^^3^
Monoclinic, *P*2_1_/*c*	Mo *K*α radiation, λ = 0.71073 Å
Hall symbol: -P 2ybc	Cell parameters from 5904 reflections
*a* = 5.2668 (1) Å	θ = 2.3–32.5°
*b* = 13.0861 (2) Å	µ = 0.26 mm^−^^1^
*c* = 18.5373 (3) Å	*T* = 100 K
β = 105.640 (1)°	Block, brown
*V* = 1230.32 (4) Å^3^	0.39 × 0.22 × 0.18 mm
*Z* = 4	

### Data collection

**Table:**

Bruker SMART APEXII CCD diffractometer	4470 independent reflections
Radiation source: fine-focus sealed tube	3657 reflections with *I* > 2σ(*I*)
Graphite monochromator	*R*_int_ = 0.036
φ and ω scans	θ_max_ = 32.6°, θ_min_ = 2.3°
Absorption correction: multi-scan (*SADABS*; Bruker, 2009)	*h* = −7→7
*T*_min_ = 0.907, *T*_max_ = 0.956	*k* = −19→19
17425 measured reflections	*l* = −26→28

### Refinement

**Table:**

Refinement on *F*^2^	Primary atom site location: structure-invariant direct methods
Least-squares matrix: full	Secondary atom site location: difference Fourier map
*R*[*F*^2^ > 2σ(*F*^2^)] = 0.047	Hydrogen site location: inferred from neighbouring sites
*wR*(*F*^2^) = 0.108	H-atom parameters constrained
*S* = 1.06	*w* = 1/[σ^2^(*F*_o_^2^) + (0.0389*P*)^2^ + 0.749*P*] where *P* = (*F*_o_^2^ + 2*F*_c_^2^)/3
4470 reflections	(Δ/σ)_max_ = 0.001
172 parameters	Δρ_max_ = 0.48 e Å^−^^3^
0 restraints	Δρ_min_ = −0.28 e Å^−^^3^

### Special details

**Table:**

Experimental. The crystal was placed in the cold stream of an Oxford Cryosystems Cobra open-flow nitrogen cryostat (Cosier & Glazer, 1986) operating at 100.0 (1) K.
Geometry. All esds (except the esd in the dihedral angle between two l.s. planes) are estimated using the full covariance matrix. The cell esds are taken into account individually in the estimation of esds in distances, angles and torsion angles; correlations between esds in cell parameters are only used when they are defined by crystal symmetry. An approximate (isotropic) treatment of cell esds is used for estimating esds involving l.s. planes.
Refinement. Refinement of F^2^ against ALL reflections. The weighted R-factor wR and goodness of fit S are based on F^2^, conventional R-factors R are based on F, with F set to zero for negative F^2^. The threshold expression of F^2^ > 2sigma(F^2^) is used only for calculating R-factors(gt) etc. and is not relevant to the choice of reflections for refinement. R-factors based on F^2^ are statistically about twice as large as those based on F, and R- factors based on ALL data will be even larger.

### Fractional atomic coordinates and isotropic or equivalent isotropic displacement parameters (Å^2^)

**Table:**

	*x*	*y*	*z*	*U*_iso_*/*U*_eq_	
S1	0.40765 (6)	0.66213 (3)	−0.105525 (19)	0.01874 (9)	
O1	0.7884 (2)	0.80307 (8)	−0.04709 (6)	0.0259 (2)	
N1	0.6697 (2)	0.49519 (9)	−0.06570 (6)	0.0169 (2)	
N2	0.9062 (2)	0.64044 (8)	−0.01161 (6)	0.0163 (2)	
H1	1.0220	0.6004	0.0140	0.020*	
C1	0.2553 (3)	0.54707 (11)	−0.13559 (8)	0.0199 (3)	
H1A	0.0793	0.5399	−0.1659	0.024*	
C2	0.4222 (3)	0.46858 (11)	−0.10991 (8)	0.0189 (3)	
H2A	0.3726	0.3994	−0.1215	0.023*	
C3	0.6867 (2)	0.59466 (10)	−0.05809 (7)	0.0154 (2)	
C4	0.9383 (3)	0.74460 (10)	−0.00491 (8)	0.0178 (2)	
C5	1.1574 (3)	0.77925 (11)	0.06208 (8)	0.0190 (3)	
H5A	1.2188	0.8483	0.0529	0.023*	
H5B	1.3087	0.7316	0.0705	0.023*	
C6	1.0495 (2)	0.78093 (10)	0.13046 (8)	0.0174 (2)	
C7	1.1084 (3)	0.70263 (11)	0.18172 (8)	0.0191 (3)	
H7A	1.2283	0.6509	0.1761	0.023*	
C8	0.9946 (3)	0.69737 (11)	0.24260 (8)	0.0211 (3)	
H8A	1.0395	0.6428	0.2775	0.025*	
C9	0.8202 (3)	0.77057 (11)	0.25133 (8)	0.0210 (3)	
H9A	0.7405	0.7655	0.2915	0.025*	
C10	0.7573 (2)	0.85443 (10)	0.20059 (8)	0.0178 (2)	
C11	0.5793 (3)	0.93187 (11)	0.20968 (8)	0.0220 (3)	
H11A	0.5005	0.9276	0.2500	0.026*	
C12	0.5200 (3)	1.01268 (11)	0.16105 (8)	0.0226 (3)	
H12A	0.3992	1.0636	0.1674	0.027*	
C13	0.6382 (3)	1.02017 (11)	0.10154 (8)	0.0218 (3)	
H13A	0.5987	1.0769	0.0684	0.026*	
C14	0.8101 (3)	0.94631 (10)	0.09084 (8)	0.0199 (3)	
H14A	0.8875	0.9525	0.0503	0.024*	
C15	0.8737 (2)	0.86059 (10)	0.13969 (7)	0.0168 (2)	

### Atomic displacement parameters (Å^2^)

**Table:**

	*U*^11^	*U*^22^	*U*^33^	*U*^12^	*U*^13^	*U*^23^
S1	0.01559 (14)	0.01887 (16)	0.02027 (16)	0.00490 (11)	0.00230 (11)	0.00192 (12)
O1	0.0310 (6)	0.0161 (5)	0.0268 (5)	0.0046 (4)	0.0013 (4)	0.0015 (4)
N1	0.0141 (5)	0.0160 (5)	0.0192 (5)	0.0005 (4)	0.0021 (4)	−0.0008 (4)
N2	0.0140 (5)	0.0131 (5)	0.0203 (5)	0.0017 (4)	0.0018 (4)	0.0008 (4)
C1	0.0138 (5)	0.0244 (7)	0.0196 (6)	0.0000 (5)	0.0013 (4)	−0.0008 (5)
C2	0.0146 (5)	0.0209 (6)	0.0197 (6)	−0.0015 (5)	0.0020 (4)	−0.0016 (5)
C3	0.0132 (5)	0.0161 (6)	0.0165 (5)	0.0016 (4)	0.0034 (4)	0.0007 (4)
C4	0.0180 (6)	0.0151 (6)	0.0214 (6)	0.0007 (4)	0.0073 (5)	−0.0011 (5)
C5	0.0170 (6)	0.0163 (6)	0.0240 (7)	−0.0008 (4)	0.0063 (5)	−0.0028 (5)
C6	0.0142 (5)	0.0159 (6)	0.0210 (6)	−0.0018 (4)	0.0027 (4)	−0.0031 (5)
C7	0.0168 (6)	0.0158 (6)	0.0223 (6)	−0.0002 (4)	0.0013 (5)	−0.0019 (5)
C8	0.0213 (6)	0.0187 (6)	0.0201 (6)	−0.0015 (5)	0.0002 (5)	0.0016 (5)
C9	0.0221 (6)	0.0214 (7)	0.0185 (6)	−0.0030 (5)	0.0037 (5)	−0.0016 (5)
C10	0.0147 (5)	0.0176 (6)	0.0194 (6)	−0.0028 (4)	0.0014 (4)	−0.0049 (5)
C11	0.0210 (6)	0.0214 (7)	0.0238 (7)	−0.0010 (5)	0.0063 (5)	−0.0063 (5)
C12	0.0209 (6)	0.0178 (6)	0.0274 (7)	0.0017 (5)	0.0035 (5)	−0.0057 (5)
C13	0.0236 (6)	0.0137 (6)	0.0254 (7)	0.0016 (5)	0.0021 (5)	−0.0019 (5)
C14	0.0210 (6)	0.0162 (6)	0.0221 (6)	−0.0012 (5)	0.0052 (5)	−0.0018 (5)
C15	0.0145 (5)	0.0155 (6)	0.0189 (6)	−0.0018 (4)	0.0019 (4)	−0.0029 (4)

### Geometric parameters (Å, º)

**Table:**

S1—C1	1.7264 (15)	C7—C8	1.415 (2)
S1—C3	1.7367 (13)	C7—H7A	0.9500
O1—C4	1.2197 (17)	C8—C9	1.367 (2)
N1—C3	1.3098 (17)	C8—H8A	0.9500
N1—C2	1.3846 (17)	C9—C10	1.425 (2)
N2—C4	1.3749 (17)	C9—H9A	0.9500
N2—C3	1.3786 (16)	C10—C11	1.4209 (19)
N2—H1	0.8458	C10—C15	1.4247 (19)
C1—C2	1.3523 (19)	C11—C12	1.370 (2)
C1—H1A	0.9500	C11—H11A	0.9500
C2—H2A	0.9500	C12—C13	1.409 (2)
C4—C5	1.5188 (19)	C12—H12A	0.9500
C5—C6	1.5229 (19)	C13—C14	1.375 (2)
C5—H5A	0.9900	C13—H13A	0.9500
C5—H5B	0.9900	C14—C15	1.4238 (19)
C6—C7	1.3747 (19)	C14—H14A	0.9500
C6—C15	1.4350 (18)		
			
C1—S1—C3	88.65 (6)	C6—C7—H7A	119.3
C3—N1—C2	109.86 (11)	C8—C7—H7A	119.3
C4—N2—C3	123.32 (11)	C9—C8—C7	120.21 (13)
C4—N2—H1	120.7	C9—C8—H8A	119.9
C3—N2—H1	116.0	C7—C8—H8A	119.9
C2—C1—S1	110.32 (10)	C8—C9—C10	120.38 (13)
C2—C1—H1A	124.8	C8—C9—H9A	119.8
S1—C1—H1A	124.8	C10—C9—H9A	119.8
C1—C2—N1	115.87 (13)	C11—C10—C15	119.47 (13)
C1—C2—H2A	122.1	C11—C10—C9	120.96 (13)
N1—C2—H2A	122.1	C15—C10—C9	119.57 (12)
N1—C3—N2	121.37 (11)	C12—C11—C10	120.89 (14)
N1—C3—S1	115.27 (10)	C12—C11—H11A	119.6
N2—C3—S1	123.24 (10)	C10—C11—H11A	119.6
O1—C4—N2	121.42 (13)	C11—C12—C13	119.88 (13)
O1—C4—C5	123.70 (13)	C11—C12—H12A	120.1
N2—C4—C5	114.69 (12)	C13—C12—H12A	120.1
C4—C5—C6	108.24 (11)	C14—C13—C12	120.76 (14)
C4—C5—H5A	110.1	C14—C13—H13A	119.6
C6—C5—H5A	110.1	C12—C13—H13A	119.6
C4—C5—H5B	110.1	C13—C14—C15	120.87 (13)
C6—C5—H5B	110.1	C13—C14—H14A	119.6
H5A—C5—H5B	108.4	C15—C14—H14A	119.6
C7—C6—C15	119.46 (13)	C14—C15—C10	118.10 (12)
C7—C6—C5	119.95 (12)	C14—C15—C6	123.03 (13)
C15—C6—C5	120.50 (12)	C10—C15—C6	118.87 (12)
C6—C7—C8	121.48 (13)		
			
C3—S1—C1—C2	−1.29 (11)	C7—C8—C9—C10	−1.9 (2)
S1—C1—C2—N1	0.83 (16)	C8—C9—C10—C11	−179.05 (13)
C3—N1—C2—C1	0.34 (18)	C8—C9—C10—C15	1.2 (2)
C2—N1—C3—N2	174.69 (12)	C15—C10—C11—C12	−0.5 (2)
C2—N1—C3—S1	−1.39 (15)	C9—C10—C11—C12	179.70 (13)
C4—N2—C3—N1	176.49 (13)	C10—C11—C12—C13	−0.7 (2)
C4—N2—C3—S1	−7.74 (18)	C11—C12—C13—C14	1.0 (2)
C1—S1—C3—N1	1.59 (11)	C12—C13—C14—C15	−0.2 (2)
C1—S1—C3—N2	−174.41 (12)	C13—C14—C15—C10	−1.0 (2)
C3—N2—C4—O1	−9.5 (2)	C13—C14—C15—C6	179.30 (13)
C3—N2—C4—C5	165.66 (12)	C11—C10—C15—C14	1.30 (18)
O1—C4—C5—C6	92.07 (16)	C9—C10—C15—C14	−178.91 (12)
N2—C4—C5—C6	−82.97 (14)	C11—C10—C15—C6	−178.95 (12)
C4—C5—C6—C7	101.68 (14)	C9—C10—C15—C6	0.84 (18)
C4—C5—C6—C15	−74.62 (15)	C7—C6—C15—C14	177.58 (12)
C15—C6—C7—C8	1.5 (2)	C5—C6—C15—C14	−6.11 (19)
C5—C6—C7—C8	−174.79 (12)	C7—C6—C15—C10	−2.16 (18)
C6—C7—C8—C9	0.5 (2)	C5—C6—C15—C10	174.15 (11)

### Hydrogen-bond geometry (Å, º)

**Table:**

*D*—H···*A*	*D*—H	H···*A*	*D*···*A*	*D*—H···*A*
N2—H1···N1^i^	0.85	2.07	2.9099 (16)	172
C13—H13*A*···O1^ii^	0.95	2.52	3.1929 (19)	128

Symmetry codes: (i) −*x*+2, −*y*+1, −*z*; (ii) −*x*+1, −*y*+2, −*z*.
